# Alcohol consumption and incident atrial fibrillation: rethinking dose, risk, and dogma

**DOI:** 10.1093/europace/euag029

**Published:** 2026-02-25

**Authors:** Marco Zuin, Matteo Bertini, Giuseppe Boriani

**Affiliations:** Department of Translational Medicine, University of Ferrara, Via Luigi Borsari 46, Ferrara 44100, Italy; Department of Cardio-Thoraco-Vascular Sciences and Public Health, University of Padova, Padua 35128, Italy; PhD Program in Translation Specialistic Medicine ‘G.B. Morgagni’, Curriculum Cardiovascular Sciences, University of Padua, Padua 35128, Italy; Department of Cardiology, Madre Teresa di Calcutta Hospital, AULSS 6, South Padova Hospitals, Schiavonia 35043, Italy; Department of Translational Medicine, University of Ferrara, Via Luigi Borsari 46, Ferrara 44100, Italy; Cardiology Unit, Department of Translational Medicine, University of Ferrara, Ferrara, Italy; Cardiology Division, Department of Biomedical, Metabolic and Neural Sciences, University of Modena and Reggio Emilia, Policlinico di Modena, Modena, Italy

**Keywords:** Atrial fibrillation, Alcohol, Comorbidities, Meta-analysis, Risk factors

**This editorial refers to ‘Alcohol consumption and risk of atrial fibrillation: a pairwise and network meta-analysis’ by A.A. Zain Ul *et al.*, https://doi.org/10.1093/europace/euag111.**

The association between alcohol consumption and atrial fibrillation (AF) has long been considered straightforward: Increasing intake confers increasing risk.^1,2^ This assumption has shaped previous and current guideline recommendations as well as public health messaging, and routine clinical counselling. However, over the last years, accumulating epidemiological evidence has increasingly challenged this linear model. In this context, the comprehensive meta-analysis by Zain Ul *et al*.,^3^ published in this issue of *Europace*, represents a timely and methodologically rigorous reappraisal of the alcohol–AF relationship.

**Figure 1:**
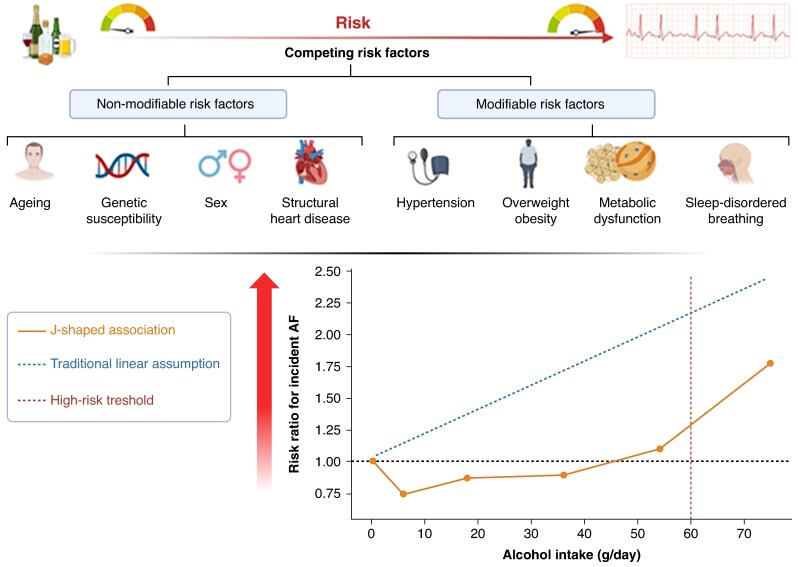
Alcohol consumption and incident atrial fibrillation within a competing risk factor framework. The schematic illustrates the relationship between alcohol intake and incident atrial fibrillation (AF) in the context of non-modifiable and modifiable competing risk factors. The lower panel depicts the traditional assumption of a linear increase in AF risk with rising alcohol consumption (dashed line) contrasted with contemporary evidence supporting a non-linear, J-shaped association (continuous line). Low to moderate alcohol intake appears neutral or inversely associated with AF risk compared with abstinence, whereas high intake is associated with a marked increase in risk. The vertical dashed line indicates a proposed high-risk threshold (∼≥60 g/day), beyond which AF risk rises steeply. The upper panel highlights major non-modifiable (ageing, genetic susceptibility, sex, structural heart disease) and modifiable (hypertension, overweight/obesity, metabolic dysfunction, sleep-disordered breathing) factors that compete with and modify the alcohol–AF relationship, underscoring the need for individualized risk assessment within a broader cardiovascular prevention framework.

By integrating pairwise meta-analysis, meta-regression, dose–response modelling, and network meta-analysis across nearly 15 million individuals, the authors provide the most granular assessment to date of how differing levels of alcohol exposure relate to incident AF. Their findings suggest a non-linear, J-shaped association, with very high alcohol intake (>60 g/day) clearly associated with excess AF risk, while low and moderate intake appear neutral or even inversely associated when compared with abstinence^3^ (*Figure 1*).

A key strength of this work lies in the standardization of alcohol exposure into pre-specified gram-per-day categories. This approach addresses one of the major limitations of prior investigations, where heterogeneous and often arbitrary definitions of ‘light’, ‘moderate’, and ‘heavy’ drinking impeded meaningful comparisons. Moreover, the use of network meta-analysis further allows indirect comparisons across multiple exposure strata, highlighting potential threshold effects that are not easily captured in conventional pairwise analyses.

At the same time, the interpretive boundaries of observational data remain firmly in place. The absence of randomized evidence, reliance on self-reported alcohol intake, and lack of direct head-to-head comparisons between non-zero drinking categories necessitate caution. Additionally, the observed inverse associations at low-to-moderate intake should therefore be viewed as signals rather than proof of protection.^3^

Importantly, alcohol consumption is not merely a biochemical exposure but also a behavioural and social marker. Low or moderate intake often clusters with broader lifestyle patterns, including dietary quality, physical activity, social engagement, and healthcare access, that themselves influence AF risk. Conversely, abstinence groups may include individuals who have reduced or ceased alcohol consumption due to underlying illness, frailty, or cardiovascular risk, introducing abstainer or ‘sick quitter’ bias. These considerations are particularly relevant in AF, a condition that emerges from the cumulative burden of ageing, cardiometabolic disease, and structural atrial remodelling, referred to as atrial cardiomyopathy.^4^ In this setting, modest alcohol intake may act less as a protective factor and more as a surrogate for a lower-risk phenotype.

Another important issue highlighted by Zain Ul and coll. is the potential disconnect between alcohol's relationship with AF and its effects on upstream cardiovascular risk factors.^3^ Hypertension, obesity, and metabolic dysfunction, which are well-known key drivers of AF, are themselves adversely influenced by alcohol at relatively low thresholds.^5,6^ Recent dose–response analyses suggest that blood pressure rises begin well below the levels at which AF risk increased^7^ in this meta-analysis, raising the possibility that alcohol-related harm may manifest through longer-term structural and vascular pathways not fully captured by incident AF alone. This underscores the need to interpret alcohol–AF associations within a broader cardiovascular context rather than in isolation.^8^

From a clinical perspective, these findings invite nuance rather than revisionism. Specifically, these results do not support recommending alcohol consumption as a preventive strategy for AF, nor do they justify relaxation of existing guideline advice. Instead, they reinforce the importance of reframing alcohol counselling away from binary notions of ‘safe’ vs. ‘unsafe’ and towards a more individualized assessment of risk.

Very high alcohol intake emerges unequivocally as harmful, with a consistently elevated risk of AF that is biologically plausible and clinically actionable. Identifying and addressing heavy drinking should therefore remain a priority in daily clinical practice, particularly in individuals with additional AF risk factors or established cardiovascular disease. At the same time, the observation that risk is not uniform across the drinking spectrum highlights the limitations of one-size-fits-all messaging. For many individuals, particularly those without prior AF and without high-risk comorbidities, modest alcohol consumption may not meaningfully increase short-term AF risk, although it may still contribute to other adverse outcomes over time.

In practice, discussions around alcohol should be embedded within a comprehensive risk factor modification framework rather than treated as an isolated exposure. Integrated care models, such as the ABC and AF-CARE pathways, provide a useful structure for this approach, emphasizing holistic management of comorbidities, lifestyle factors, and patient preferences.^9^ Within such frameworks, alcohol counselling becomes part of a broader conversation about blood pressure control, weight management, sleep health, and metabolic risk, factors that collectively shape the atrial substrate and long-term AF trajectory. A recent EHRA survey showed that around 90% of cardiologist involved in AF care of routinely interview AF patients about their alcohol intake, but only 30% were actually able to refer patients with excessive alcohol intake to a dedicated alcohol reduction/cessation service in their centre.^10^

This study also raises important questions for guideline development and public health communication.^3^ While simplicity is essential for population-level messaging, oversimplification risks obscuring clinically meaningful heterogeneity. Future recommendations may benefit from greater emphasis on thresholds, patterns of consumption, and patient-specific risk profiles, rather than uniform limits alone.

Looking ahead, several priorities emerge. Prospective studies with repeated assessment of alcohol intake, detailed characterization of drinking patterns, including binge drinking, and continuous rhythm monitoring are needed to clarify causal pathways and assess AF burden rather than binary incidence.^11^ Mechanistic studies exploring the interaction between alcohol, atrial remodelling, autonomic tone, and inflammation may further illuminate why threshold effects appear to dominate risk. Finally, greater attention to sex, gender, age, and comorbidity interactions will be essential to refine personalized prevention strategies.

In conclusion, this landmark meta-analysis by Zain *et al*.^3^ reframes the relationship between alcohol consumption and incident AF as non-linear and threshold-dependent, challenging long-standing assumptions while reaffirming the harms of heavy drinking. Rather than prompting immediate changes in clinical recommendations, these findings encourage more individualized, context-aware counselling and highlight the need for a broader, integrated approach to AF prevention. In doing so, they move the field beyond dogma and towards a more nuanced understanding of lifestyle-related AF risk.
